# Curcumin-loaded chitosan thermosensitive hydrogel with sustained release for periodontal applications: an *in vitro* evaluation

**DOI:** 10.1590/1678-7765-2026-0308

**Published:** 2026-08-03

**Authors:** Reema M Rao, Monali Nikalje, Shiprita Kumari, Rahul Tiwari, Kailash Chandra Dash, Saurabh Shekhar, Manish Sharma

**Affiliations:** 1 Nitte (Deemed to be University) AB Shetty Memorial Institute of Dental Sciences Department of Periodontics Mangaluru Karnataka India Nitte (Deemed to be University), AB Shetty Memorial Institute of Dental Sciences, Department of Periodontics, Mangaluru, Karnataka, India.; 2 Symbiosis Medical College for Women Department of Dentistry Pune Maharashtra India Symbiosis Medical College for Women, Department of Dentistry, Pune, Maharashtra, India.; 3 AIMST University Bedong Kedah Malaysia AIMST University, Bedong, Kedah, Malaysia.; 4 Dr. D. Y. Patil Vidyapeeth (Deemed to be University) Dr. D. Y. Patil Dental College & Hospital Department of Dental Research Cell Pune Maharashtra India Dr. D. Y. Patil Vidyapeeth (Deemed to be University), Dr. D. Y. Patil Dental College & Hospital, Department of Dental Research Cell, Pune, Maharashtra, India.; 5 Kalinga Institute of Industrial Technology - KIIT (Deemed to be University) Kalinga Institute of Dental Sciences Department of Oral and Maxillofacial Pathology Patia Odisha India Kalinga Institute of Industrial Technology - KIIT (Deemed to be University), Kalinga Institute of Dental Sciences, Department of Oral and Maxillofacial Pathology, Patia, Odisha, India.; 6 JMF's ACPM Dental College Department of Oral Pathology Dhule Maharashtra India JMF's ACPM Dental College, Department of Oral Pathology, Dhule, Maharashtra, India.

**Keywords:** Thermosensitive hydrogel, Localized periodontal drug delivery, Sustained-release biomaterials, Antibiofilm activity, Periodontitis

## Abstract

**Objective:**

Thermosensitive hydrogels have emerged as promising localized drug delivery systems for periodontal therapy. Curcumin possesses anti-inflammatory and antimicrobial properties but is limited by its poor solubility and bioavailability. This study aimed to develop and evaluate a curcumin-loaded chitosan thermosensitive hydrogel for periodontal application.

**Methodology:**

This *in vitro* experimental study included five groups as positive controls, negative control, blank chitosan hydrogel, free curcumin, curcumin-loaded hydrogel, and chlorhexidine (0.12%). The hydrogel was formulated using chitosan and β-glycerophosphate and was characterized for thermosensitive gelation and injectability. Drug release was assessed using dialysis. Cytocompatibility was evaluated in human gingival fibroblasts (HGF-1) using the MTT assay at 24, 48, and 72 h. The anti-inflammatory activity was assessed by measuring the levels of TNF-α and IL-1β in LPS-stimulated cells. Antibacterial activity (MIC/MBC) and antibiofilm efficacy were tested against *Porphyromonas gingivalis* and *Aggregatibacter actinomycetemcomitans*. Data were analyzed using ANOVA with the appropriate post-hoc tests (p<0.05).

**Results:**

The curcumin-loaded hydrogel showed high cell viability (>90%) and significantly reduced TNF-α and IL-1β levels when compared with free curcumin. It demonstrated improved antibiofilm activity (∼74–79%) and lower MIC values than free curcumin, although slightly less effective than chlorhexidine. A sustained drug release profile (∼81.6% at 14 days) following Korsmeyer–Peppas kinetics was observed.

**Conclusion:**

The curcumin-loaded chitosan thermosensitive hydrogel showed improved biological performance and sustained release when compared with free curcumin, suggesting its potential as a localized periodontal drug delivery system. Further *in vivo* studies are required to validate these findings.

## Graphical abstract

**Figure:**
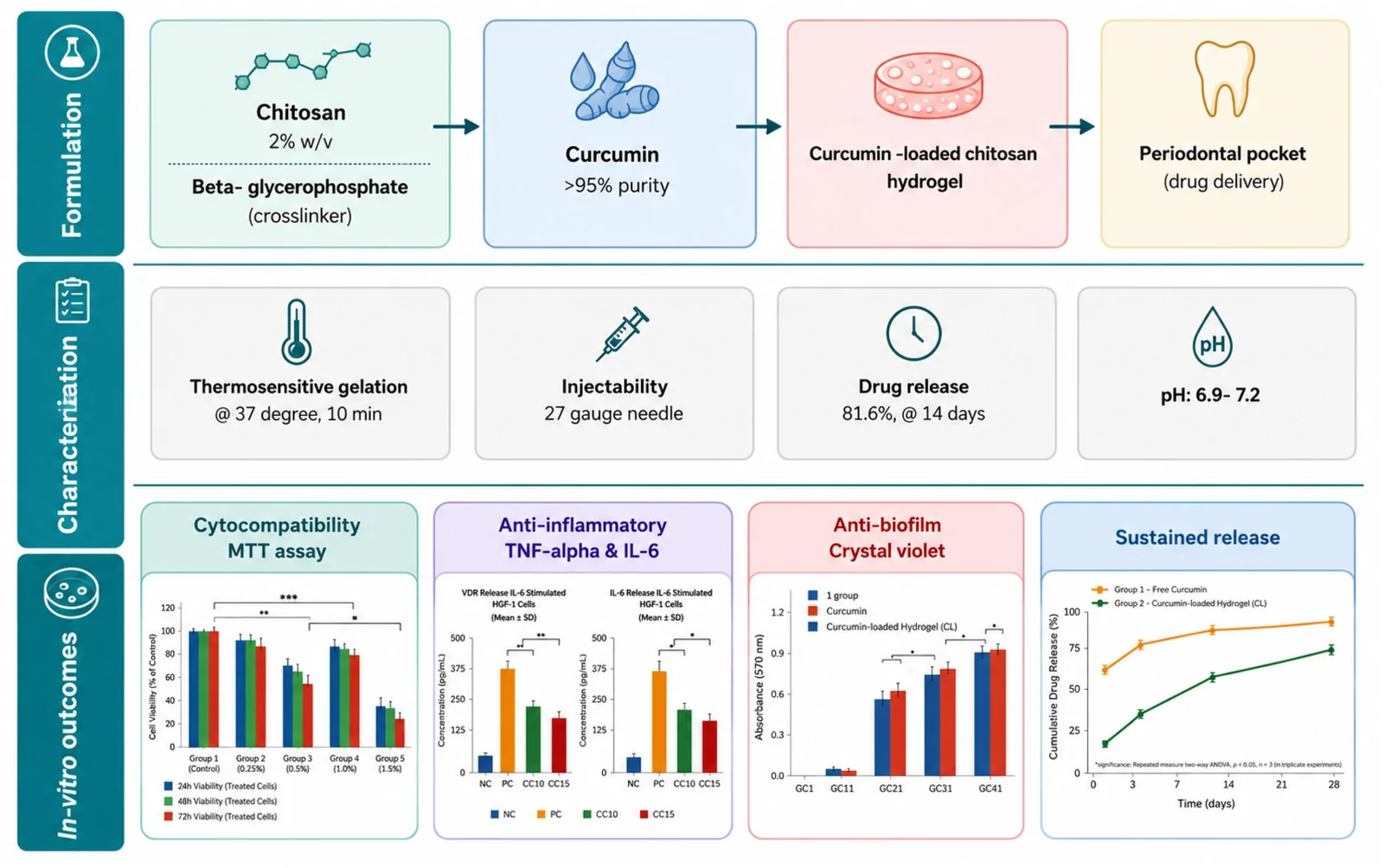

## Introduction

Periodontal disease is a chronic inflammatory condition characterized by the progressive destruction of the supporting tissues of teeth, primarily driven by pathogenic biofilms and an exacerbated host immune response.^1^ Conventional periodontal therapy, including scaling and root planing combined with systemic or local antimicrobials, often shows limited long-term efficacy owing to poor drug retention at the site, systemic side effects, and an inability to sustain therapeutic concentrations within periodontal pockets.^2,3^

Localized drug delivery systems have emerged as a promising strategy to overcome these limitations by enabling the site-specific controlled release of therapeutic agents. Among these, thermosensitive hydrogels have gained considerable attention because of their injectable nature, *in situ* gelation at physiological temperatures, and ability to prolong drug residence time.^4,5^ Chitosan-based hydrogels offer additional advantages such as biocompatibility, biodegradability, mucoadhesion, and inherent antimicrobial properties.^6^ In thermosensitive hydrogel systems, chitosan plays an important role as a biopolymeric matrix capable of undergoing sol-to-gel transition in the presence of suitable agents such as β-glycerophosphate. Its cationic and mucoadhesive properties enhance retention within periodontal pockets and facilitate prolonged local drug release. Furthermore, the porous polymeric network formed by chitosan contributes to the sustained delivery of incorporated therapeutic agents while maintaining favorable tissue compatibility.

Curcumin, a natural polyphenolic compound derived from *Curcuma longa*, shows potent anti-inflammatory, antioxidant, and antimicrobial properties.^7^ Despite its potential in periodontal therapy, its clinical application is significantly hindered by poor aqueous solubility, rapid degradation, and low bioavailability.^8^ Therefore, incorporating curcumin into a suitable carrier system is essential to enhance its stability and therapeutic efficacy.

Previous studies have independently explored chitosan-based hydrogels and curcumin for periodontal applications and highlighted the potential of multifunctional hydrogel systems incorporating bioactive agents for periodontal applications.^5,6,9,10^ For example, the supramolecular hydrogel CaF2 periodontal study by Zhang, et al.^11^ (2024) have shown that a supramolecular composite hydrogel loaded with calcium fluoride nanoparticles significantly enhanced periodontal bone regeneration, underscoring the therapeutic potential of advanced hydrogel-based delivery platforms. However, such systems primarily focus on regenerative outcomes and nanoparticle incorporation, with a comparatively limited emphasis on the simultaneous modulation of inflammation, microbial load, and biofilm formation using naturally derived bioactive compounds.

Recent studies have further highlighted the therapeutic potential of curcumin-based delivery systems in oral and regenerative medicine. Diomede, et al.^12^ (2021) have shown that liposomal curcumin effectively modulated inflammatory responses and cellular morphology in lipopolysaccharide-stimulated endothelial committed neural crest-derived stem cells, supporting its potential role in periodontal inflammatory conditions. Similarly, Sinjari, et al.^13^ (2019) have reported that curcumin-loaded liposomal formulations exerted significant anti-inflammatory effects by modulating the NF-κB/ERK/pERK signaling pathway in human dental pulp cells. These findings emphasize the importance of advanced delivery platforms in enhancing the biological efficacy and stability of curcumin for oral therapeutic applications.

Despite growing interest in hydrogel-based systems and curcumin individually, there remains a lack of well-designed thermosensitive formulations that integrate sustained and localized drug delivery with antibacterial, antibiofilm, and anti-inflammatory effects, particularly targeting key periodontal pathogens and host cellular responses. Therefore, this study was designed to develop and evaluate a curcumin-loaded chitosan thermosensitive hydrogel for periodontal applications. This study focused on formulating and optimizing a chitosan/β-glycerophosphate-based thermosensitive system incorporating curcumin, followed by the characterization of its gelation behavior at physiological temperatures. Furthermore, its *in vitro* performance was assessed regarding drug release kinetics, cytocompatibility with human gingival fibroblasts, and anti-inflammatory activity by quantifying proinflammatory cytokines in lipopolysaccharide-stimulated cells. Additionally, the antibacterial and antibiofilm efficacies of the formulation against key periodontal pathogens were evaluated to determine its potential as a localized therapeutic system.

The need for this study arises from the increasing demand for minimally invasive, multifunctional biomaterials capable of addressing the complex pathophysiology of periodontal disease by simultaneously controlling infection, inflammation, and tissue compatibility.

## Methodology

This *in vitro* experimental study was conducted in the Department of Periodontology using standardized laboratory protocols for biomaterial evaluation. The study design followed established *in vitro* research recommendations and was aligned with the ISO 10993 guidelines for the biological evaluation of biomaterials and the Clinical and Laboratory Standards Institute guidelines for antimicrobial testing.

The experimental setup consisted of five groups: I (negative control), II (blank chitosan thermosensitive hydrogel), III (free curcumin solution), IV (curcumin-loaded chitosan thermosensitive hydrogel), and V (0.12% chlorhexidine as a positive control). These groups were evaluated across all assays to ensure a uniform comparison.

Chitosan (medium molecular weight, ≥85% degree of deacetylation; Sigma-Aldrich, St. Louis, MO, USA), β-glycerophosphate disodium salt hydrate (Sigma-Aldrich, St. Louis, MO, USA), curcumin (≥95% purity; Sigma-Aldrich, St. Louis, MO, USA), and lipopolysaccharide from *Escherichia coli* O111:B4 (Sigma-Aldrich, St. Louis, MO, USA) were used in this study. Dulbecco's modified Eagle's medium, fetal bovine serum, and penicillin-streptomycin were obtained from Gibco (Thermo Fisher Scientific, Waltham, MA, USA). Human gingival fibroblasts (HGF-1, ATCC CRL-2014), *Porphyromonas gingivalis* (ATCC 33277), and *Aggregatibacter actinomycetemcomitans* (ATCC 43718) were procured from the American Type Culture Collection (ATCC, Manassas, VA, USA). ELISA kits for TNF-α and IL-1β (R&D Systems, Minneapolis, MN, USA), MTT assay kits (Sigma-Aldrich, St. Louis, MO, USA), and crystal violet staining (HiMedia Laboratories, Mumbai, India) were used for analyses. All reagents had analytical or cell culture grade.

A chitosan solution (2% w/v) was prepared by dissolving chitosan in 0.1 M glacial acetic acid under continuous stirring, followed by pH adjustment to 6.0-6.2 using sodium hydroxide. The solution was filtered, dialyzed against distilled water for 24-48 h at 4°C, sterilized using a 0.22-μm membrane filter, and stored at 4°C until further use. A β-glycerophosphate solution was prepared in cold distilled water and added dropwise to the chilled chitosan solution under continuous stirring in an ice bath to obtain a thermosensitive system. Curcumin was incorporated into the 2% w/v chitosan solution at a final concentration of 10 mg/mL prior to the addition of β-glycerophosphate to prepare the curcumin-loaded chitosan thermosensitive hydrogel. The final formulation was adjusted to near physiological pH (6.9-7.2) and stored at 4°C in sol form until use. Blank hydrogel and free curcumin formulations were also prepared for comparison.

Thermosensitive gelation was assessed using the tube inversion method by incubating the samples at 37°C and recording the time required for the sol-to-gel transition. Injectability was qualitatively evaluated by extrusion using a 21-gauge needle, which was selected to simulate clinically relevant periodontal delivery conditions while permitting the smooth administration of the thermosensitive hydrogel formulation.^14^ *In vitro* drug release was assessed using the dialysis bag method (MWCO 12–14 kDa; Sigma-Aldrich, USA) in phosphate-buffered saline (pH 7.4) at 37°C under agitation. Samples were collected at predetermined intervals and analyzed using a UV–visible spectrophotometer (UV-1800, Shimadzu, Kyoto, Japan) at 425 nm. Release kinetics were evaluated using standard mathematical models.

For cytotoxicity assessment, human gingival fibroblasts were cultured in Dulbecco's modified Eagle's medium supplemented with 10% fetal bovine serum and 1% antibiotics at 37°C in a humidified 5% CO2 atmosphere. Cells were seeded in 96-well plates and exposed to the extracts from all groups for 24, 48, and 72 h. Cell viability was determined using the MTT assay, and absorbance was measured using a microplate reader (Bio-Rad Laboratories, Hercules, CA, USA). The results are shown relative to untreated controls. Anti-inflammatory activity was evaluated by stimulating fibroblasts with lipopolysaccharide (1 μg/mL), followed by treatment with the test formulations. Culture supernatants were collected, and TNF-α and IL-1β levels were quantified using ELISA kits according to the manufacturer's instructions.

Antibacterial activity was determined using broth microdilution in accordance with the Clinical and Laboratory Standards Institute guidelines to establish the minimum inhibitory and bactericidal concentrations against *P. gingivalis* and *A. actinomycetemcomitans*. Antibiofilm activity was evaluated using crystal violet staining, wherein preformed biofilms were treated with test formulations, stained, and spectrophotometrically quantified.

The sample size was calculated on G*Power (version 3.1.9.7; Heinrich Heine University, Düsseldorf, Germany) based on a one-way ANOVA model with five groups (effect size 0.40, α = 0.05, power = 80%). In total, six replicates per group per time point were used for cell viability assays, four for cytokine and antibiofilm assays, and three for drug release and gelation studies.

Statistical analyses were performed on IBM SPSS Statistics (version 25.0; IBM Corp., Armonk, NY, USA). Data are shown as mean ± standard deviation. Normality was assessed using the Shapiro–Wilk test and homogeneity of variance by the Levene's test. Two-way analysis of variance was applied for cell viability to assess the effects of group and time, whereas one-way ANOVA was used for cytokine levels and antibiofilm activity. Post-hoc comparisons were performed using Bonferroni and Tukey's tests, as appropriate. Gelation time was analyzed using an independent sample t-test. Statistical significance was set at *p*<0.05.

## Results

The results demonstrated statistically significant differences between the experimental groups across all evaluated parameters, including cytocompatibility, anti-inflammatory response, antimicrobial activity, antibiofilm efficacy, and drug-release behavior.

Cell viability analysis revealed a significant effect of treatment group and time interval (*p*<0.001), whereas the interaction between group and time was not significant. The curcumin-loaded hydrogel and blank chitosan hydrogel maintained consistently high cell viability across all time points, comparable to that of the negative control. In contrast, free curcumin exhibited a gradual decline in viability over time, with the chlorhexidine group showing the lowest viability values. Post-hoc comparisons confirmed significantly reduced viability in the chlorhexidine and free curcumin groups when compared to that in the hydrogel groups (Table 1; Figure 1a).

**Table 1:** Cell viability (%) of human gingival fibroblasts at different time intervals.

Group	24 hours			48 hours			72 hours		
	Mean (%)	SD	SEM	Mean (%)	SD	SEM	Mean (%)	SD	SEM
Group I: Negative Control	100.0	0.00	0.00	100.0	0.00	0.00	100.0	0.00	0.00
Group II: Blank Chitosan Hydrogel	97.2	1.90	0.78	95.8	2.30	0.94	94.1	2.50	1.02
Group III: Free Curcumin	87.3	2.60	1.06	83.6	3.10	1.27	79.8	3.40	1.39
Group IV: Curcumin-loaded chitosan thermosensitive hydrogel	95.1	2.10	0.86	93.7	2.40	0.98	91.5	2.70	1.10
Group V: Positive Control (CHX)	72.4	3.10	1.27	71.0	3.50	1.43	68.3	3.80	1.55
Group	F = 386.33					*p* < 0.001*			
Time	F = 15.66					*p* < 0.001*			
Group x time	F = 1.687					*p* = 0.116			

Values are shown as Mean ± SD (n = 6 per group per time point). Group I was considered as 100% reference. Two-way ANOVA showed significant effects of group and time (*p* < 0.001). Cell viability ≥70% is considered non-cytotoxic as per ISO 10993-5, SEM: Standard error of the mean

**Figure 1:**
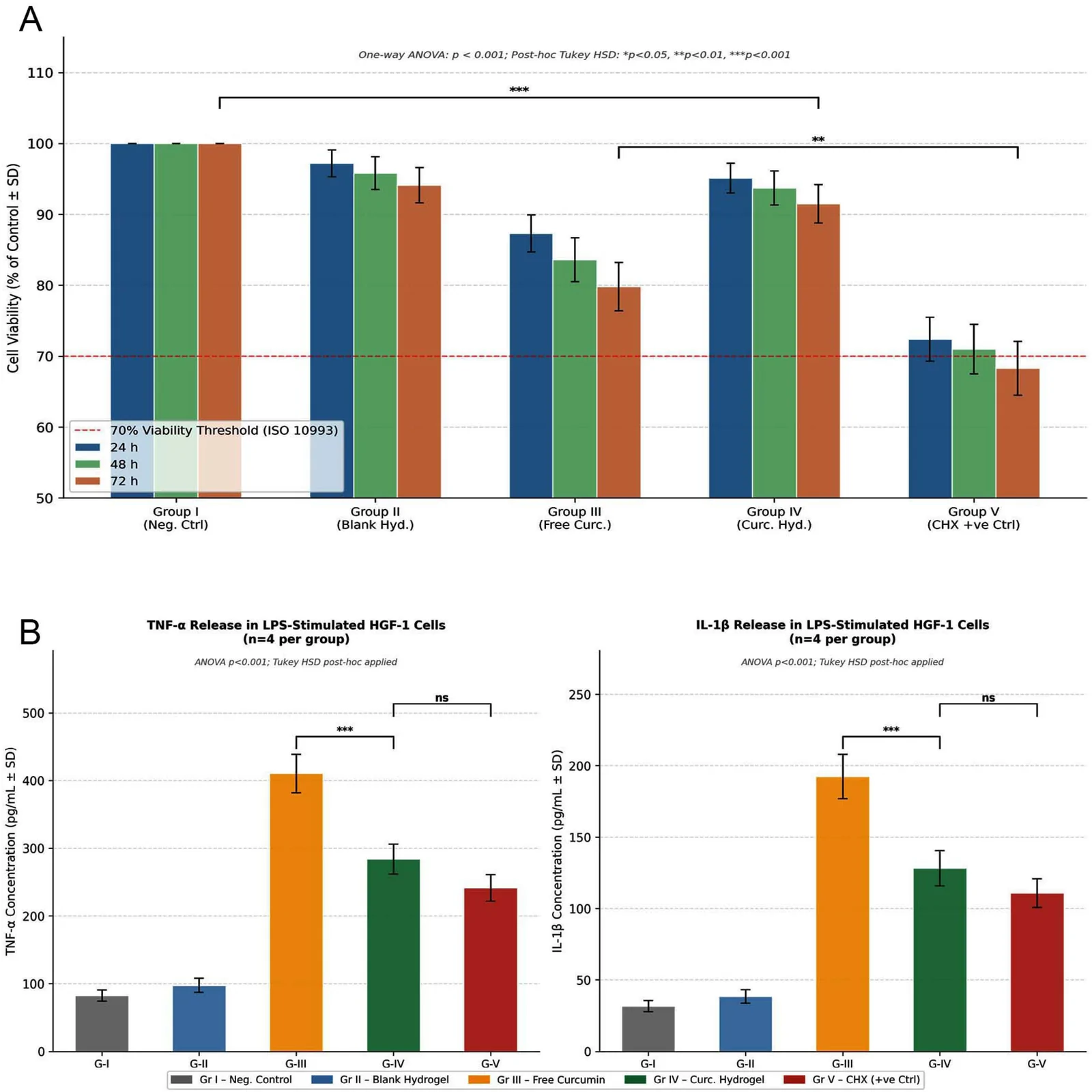
Cytocompatibility and anti-inflammatory response of test formulations. **(A)** Cell viability (%) of human gingival fibroblasts (HGF-1) following exposure to test formulations at 24, 48, and 72 hours, assessed using the MTT assay. **(B)** Pro-inflammatory cytokine levels (TNF-α and IL-1β) in LPS-stimulated HGF-1 cells following treatment with test formulations, quantified using ELISA.

*p<0.05, p<0.01, p<0.001* indicate statistical significance; ns: not significant. n=6 for cell viability and n=4 for cytokine assays. CHX: chlorhexidine; LPS: lipopolysaccharide; HGF-1: human gingival fibroblasts.

The analysis of pro-inflammatory cytokines showed significant intergroup differences (*p*<0.001). The curcumin-loaded hydrogel markedly reduced TNF-α and IL-1β levels when compared with free curcumin, indicating improved anti-inflammatory efficacy. The reduction by the hydrogel formulation was comparable to that achieved with chlorhexidine. In contrast, the negative control and blank hydrogel groups maintained low baseline cytokine levels, indicating minimal inflammatory responses (Table 2; Figure 1b).

**Table 2:** Pro-inflammatory cytokine levels (TNF-α and IL-1β) in LPS-stimulated cells.

Group	TNF-α (pg/mL)			F value	*p*-value	IL-1β (pg/mL)			F value	*p*-value
	Mean	SD	SEM			Mean	SD	SEM		
Group I: Negative Control	82.4	8.2	4.1	312.6	< 0.001*	31.6	3.9	2.0	287.4	< 0.001*
Group II: Blank Chitosan Hydrogel	97.3	10.4	5.2			38.5	4.7	2.4		
Group III: Free Curcumin	410.5	28.3	14.2			192.4	15.6	7.8		
Group IV: Curcumin-loaded chitosan thermosensitive hydrogel	283.7	22.1	11.1			128.3	12.4	6.2		
Group V: Positive Control (CHX)	241.2	19.6	9.8			110.7	10.1	5.1		

Values are shown as Mean ± SD (n = 4 per group). One-way ANOVA showed significant differences between groups (*p* < 0.001). Lower cytokine levels indicate greater anti-inflammatory efficacy. Cytokine levels were measured in culture supernatants using ELISA following treatment with test formulations.

Antibiofilm evaluation revealed significant differences between the groups for both tested microorganisms (*p*<0.001). The curcumin-loaded hydrogel exhibited significantly greater biofilm inhibition than free curcumin, suggesting enhanced antibiofilm activity due to sustained drug delivery. However, its effect was weaker than that of chlorhexidine, which showed the highest level of inhibition. The blank hydrogel demonstrated minimal activity, comparable to that of the negative control (Table 3; Figure 2a).

**Table 3:** Antibiofilm activity (% biofilm inhibition) of the samples.

Biofilm inhibition (%)	*P. gingivalis*			F value	*p*-value	*A. actinomycetemcomitans*			F value	*p*-value
	Mean	SD	SEM			Mean	SD	SEM		
Group I: Negative Control	0.0	0.00	0.00	528.4	< 0.001*	0.0	0.00	0.00	614.1	< 0.001*
Group II: Blank Chitosan Hydrogel	4.2	1.60	0.80			3.8	1.30	0.65		
Group III: Free Curcumin	57.8	5.30	2.65			62.4	4.70	2.35		
Group IV: Curcumin-loaded chitosan thermosensitive hydrogel	74.3	4.80	2.40			78.9	4.20	2.10		
Group V: Positive Control (CHX)	89.1	3.20	1.60			91.5	2.80	1.40		

Values are shown as Mean ± SD (n = 4 per group). One-way ANOVA showed significant differences between groups (p < 0.001). Higher percentage indicates greater biofilm inhibition, Crystal violet staining method (n = 4 per group), Biofilm inhibition against *P. gingivalis* (ATCC 33277) and *A. actinomycetemcomitans* (ATCC 43718).

**Figure 2:**
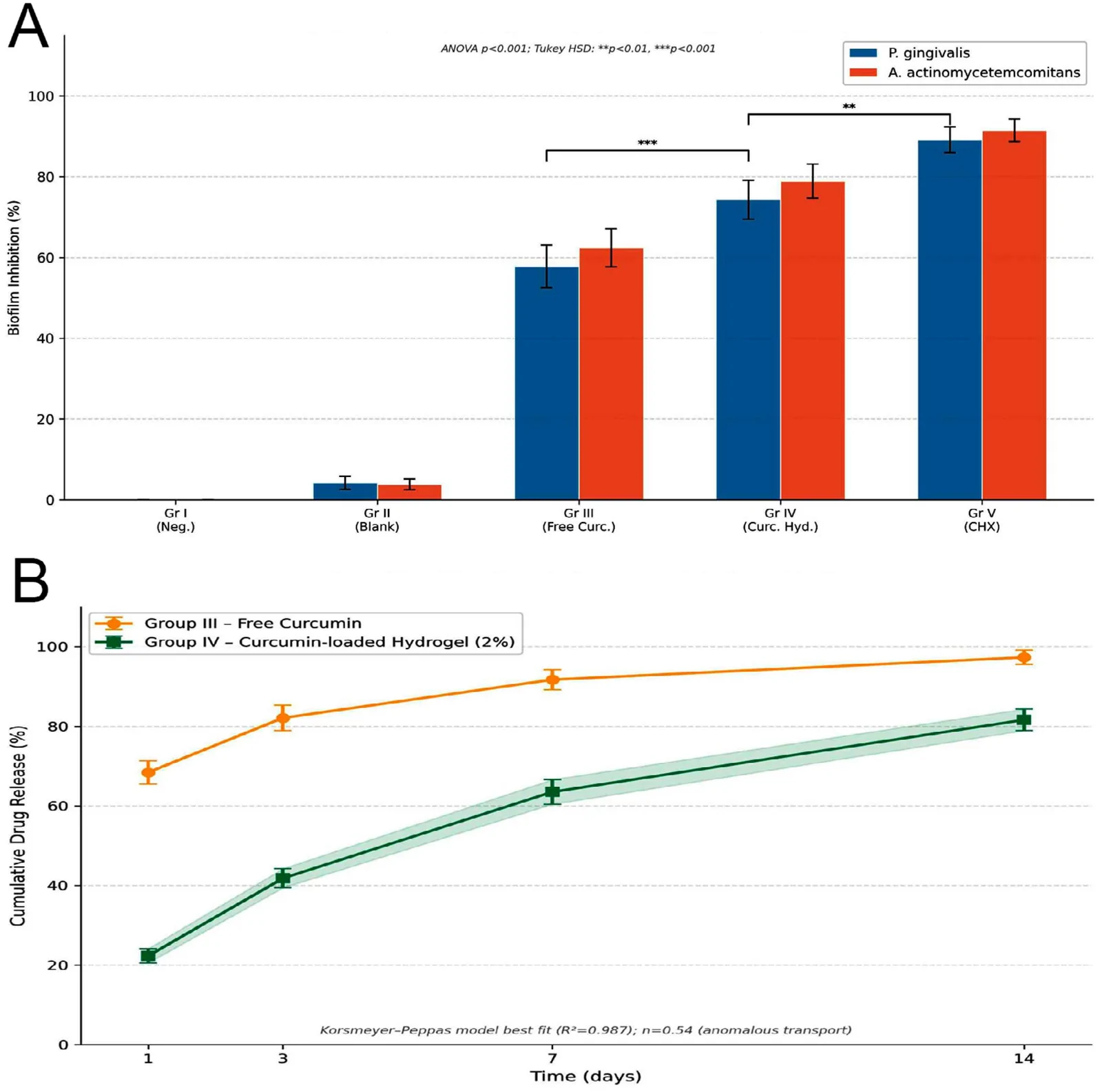
Antibiofilm activity and in vitro drug release profile of test formulations. **(A)** Antibiofilm activity (% inhibition) of test formulations against *Porphyromonas gingivalis* and *Aggregatibacter actinomycetemcomitans*, assessed using the crystal violet assay. **(B)** *In vitro* cumulative drug release (%) of curcumin from free curcumin and curcumin-loaded hydrogel in phosphate-buffered saline (pH 7.4) at 37°C over 14 days.

*p<0.05, p<0.01, p<0.001* indicate statistical significance. n=4 for antibiofilm assay and n=3 for drug release study. CHX: chlorhexidine.

The antibacterial analysis further supported these findings, with the curcumin-loaded hydrogel showing substantially improved antimicrobial efficacy when compared to free curcumin, as evidenced by the lower MIC and MBC values against both tested pathogens. The blank hydrogel and negative control showed no antibacterial activity, whereas chlorhexidine exhibited the greatest potency in all groups (Table 4).

**Table 4:** Antibacterial activity (MIC and MBC values, µg/mL) of the samples.

Group	*P. gingivalis* MIC (µg/mL)	*P. gingivalis* MBC (µg/mL)	*A. actinomycetemcomitans* MIC (µg/mL)	*A. actinomycetemcomitans*MBC (µg/mL)
Group I: Negative Control	>2048	>2048	>2048	>2048
Group II: Blank Chitosan Hydrogel	>2048	>2048	>2048	>2048
Group III: Free Curcumin	256	512	128	256
Group IV: Curcumin-loaded chitosan thermosensitive hydrogel	64	128	32	64
Group V: Positive Control (CHX)	4	8	2	4

Minimum inhibitory concentration (MIC) and minimum bactericidal concentration (MBC) were determined using broth microdilution.

This *in vitro* drug release study demonstrated distinct release patterns across the formulations. Free curcumin showed a rapid initial burst release, with near-complete release over time, whereas the curcumin-loaded hydrogel had a sustained and controlled release profile throughout the study period (Figure 2b). Kinetic modeling revealed that the release from the hydrogel system best fitted the Korsmeyer–Peppas model, followed by the Higuchi model, indicating a predominantly diffusion-controlled mechanism with a contribution from polymer matrix relaxation. The diffusion exponent suggested an anomalous (non-Fickian) transport behavior (Table 5).

**Table 5:** Drug release kinetics model fitting parameters.

Kinetic model	R^2^	K (release constant)	n (diffusion exponent)	Interpretation
Zero-Order	0.742	6.21	–	Linear; constant release rate
First-Order	0.871	–	k=0.12	Concentration-dependent release
Higuchi	0.954	23.4	–	Diffusion-controlled matrix release
Korsmeyer–Peppas	0.987	22.1	n=0.54	Best fit; anomalous (non-Fickian) transport

R^2^ ≥ 0.95 indicates good model fit. Korsmeyer–Peppas model with n = 0.54 suggests anomalous (non-Fickian) transport – combination of diffusion and erosion. Bold = best-fitting model.

Gelation studies indicated that blank and curcumin-loaded hydrogels exhibited comparable thermosensitive behavior, with no statistically significant differences in gelation time (*p* > 0.05). This suggests that incorporating curcumin did not adversely affect the gelation or injectability of the hydrogel system.

## Discussion

This study evaluated a curcumin-loaded chitosan thermosensitive hydrogel as a multifunctional local delivery system, and demonstrated consistent advantages over free curcumin regarding cytocompatibility, anti-inflammatory activity, antibiofilm efficacy, antimicrobial potency, and controlled drug release. These findings collectively support the premise that incorporating curcumin into a chitosan-based thermosensitive matrix enhances its biological performance by improving stability, retention, and bioavailability.

Regarding cytocompatibility, the blank chitosan and curcumin-loaded hydrogels maintained high cell viability when compared to the control, whereas free curcumin showed a time-dependent reduction and chlorhexidine, the lowest viability. The well-documented favorable cytocompatibility of chitosan-based systems is attributed to their biocompatible, biodegradable, and mucoadhesive nature, which minimizes cellular stress and supports cell survival.^6,15^ The reduced viability of free curcumin may be related to its poor solubility and potential for aggregation, leading to localized cytotoxic effects at higher effective concentrations.^16^ In contrast, the hydrogel matrix likely enables a more uniform and sustained release, preventing concentration spikes and improving cellular tolerance.^5^ The relatively lower cell viability of chlorhexidine is consistent with previous reports on its cytotoxic effects on human fibroblasts despite its well-established antimicrobial efficacy.^17,18^

The anti-inflammatory findings in this research further highlight the advantages of hydrogel systems. The curcumin-loaded hydrogel significantly reduced TNF-α and IL-1β levels when compared to free curcumin, with effects approaching those of chlorhexidine. Curcumin modulates inflammatory pathways, particularly by inhibiting NF-κB signaling and downregulating pro-inflammatory cytokines.^19^ However, its clinical translation is limited by poor bioavailability.^15^ Encapsulation within the chitosan hydrogel likely enhances cellular uptake and sustained exposure, amplifying its anti-inflammatory effect. Additionally, the mucoadhesive nature of chitosan may enhance the retention and interaction of the hydrogel system with gingival fibroblasts, prolonging local contact time and improving cellular exposure to curcumin. The hydrogel matrix may also protect curcumin from rapid degradation and premature diffusion, enabling sustained release and maintaining therapeutically effective concentrations over time. These combined factors could explain the greater reduction in TNF-α and IL-1β levels of the curcumin-loaded hydrogel when compared with free curcumin. Similar improvements in cytokine modulation with curcumin-loaded delivery systems have been reported in previous studies, supporting the role of controlled-release platforms in optimizing therapeutic efficacy^9,20,21^. For instance, a recent study has shown that a cellulose-based curcumin hydrogel significantly suppressed pro-inflammatory mediators such as TNF-α and IL-6, provided sustained drug release, and improved periodontal outcomes.^22^

The antibiofilm and antibacterial results in this study demonstrated a consistent pattern: the curcumin-loaded hydrogel outperformed free curcumin but remained slightly less potent than chlorhexidine. The enhanced activity of the hydrogel can be attributed to prolonged drug retention and sustained release at the target site, more effectively disrupting bacterial growth and biofilm formation. Chitosan possesses inherent antimicrobial properties, including disruption of bacterial cell membranes and interference with biofilm architecture, which may have synergistically contributed to the observed effects.^6,10,14^ The superior performance of chlorhexidine is expected owing to its broad-spectrum and rapid bactericidal action. However, its associated cytotoxicity and lack of sustained release limit its long-term applicability.^23^ The improved efficacy of the hydrogel over free curcumin is consistent with previous reports on the enhanced antimicrobial and antibiofilm activity of curcumin when delivered via nanoparticle-based and hydrogel systems.^24,25^

In contrast, the curcumin-loaded chitosan thermosensitive hydrogel demonstrated a more favorable balance between antimicrobial efficacy and cytocompatibility, with cell viability values remaining above 90% throughout the experimental period. The sustained-release characteristics of the hydrogel system may further support prolonged therapeutic action and minimize the potential adverse cellular effects associated with repeated exposure to conventional antiseptic agents such as chlorhexidine. Therefore, although chlorhexidine may provide stronger short-term antimicrobial action, hydrogel formulations may represent a more biologically compatible long-term adjunctive strategy for chronic periodontal management.

The drug release profile further substantiated the functional advantages of the hydrogel system. While free curcumin exhibited a rapid burst release, the hydrogel provided sustained and controlled release over an extended period. Kinetic modeling indicated that the release followed the Korsmeyer–Peppas model with anomalous (non-Fickian) transport, suggesting a combined mechanism of diffusion and polymer matrix relaxation.^26^ Such a release profile is particularly desirable in periodontal therapy, in which prolonged drug availability within periodontal pockets is essential for effectively managing infection and inflammation. Similar diffusion-controlled release patterns have been reported for chitosan-based hydrogels, reinforcing their suitability as local drug delivery systems.^23,27^

A previous study has also shown the effectiveness of chitosan-based nanocomposite systems in achieving sustained antimicrobial drug release and improved therapeutic performance, supporting the applicability of chitosan-derived biomaterials as controlled local delivery platforms.^28^ Further morphological characterization by scanning electron microscope may help to establish a clearer correlation between the internal porous microstructure of the hydrogel matrix and the observed sustained-release kinetics.

Importantly, the gelation assessment in this study confirmed that incorporating curcumin did not significantly alter the thermosensitive behavior of the hydrogel. Maintenance of appropriate gelation characteristics is critical for clinical applicability as it ensures ease of injection and *in situ* gel formation at physiological temperatures, enabling effective retention within periodontal pockets.

From a clinical perspective, the findings in this research suggest that a curcumin-loaded chitosan thermosensitive hydrogel could serve as a promising adjunct to conventional periodontal therapy by providing localized, sustained delivery with combined anti-inflammatory and antimicrobial effects and maintaining favorable biocompatibility. This approach may help overcome the limitations associated with conventional agents, such as rapid clearance, systemic exposure, and cytotoxicity.

This study has certain limitations. *In vitro* investigations are unable to fully replicate the complex biological environment of periodontal tissues, including host immune responses, saliva dynamics, and mechanical forces. Additionally, advanced physicochemical characterization, including rheological evaluation, scanning electron microscope-based morphological analysis, Fourier-transform infrared spectroscopy characterization, and long-term degradation studies, were not performed. These analyses could provide further insight into the internal architecture, mechanical behavior, molecular interactions, and stability of the developed hydrogel system. This study also evaluated a limited range of curcumin concentrations, and did not assess the *in vivo* efficacy or clinical outcomes.

Future studies should focus on *in vivo* and clinical investigations to validate the therapeutic potential of this system under physiological conditions. Further optimization of formulation parameters, incorporation of additional bioactive agents, and evaluation of long-term stability and degradation behavior would strengthen translational applicability. Advanced physicochemical characterization, including rheological evaluation, scanning electron microscope-based morphological analysis, and Fourier-transform infrared spectroscopy, should also be performed to better understand the structural and mechanical properties of the hydrogel system. Comparative studies with commercially available local drug delivery systems may further provide insights into their clinical applicability, therapeutic superiority, and cost-effectiveness.

## Conclusion

Within the limitations of this *in vitro* study, the curcumin-loaded chitosan thermosensitive hydrogel demonstrated improved performance compared with free curcumin across multiple parameters, including cytocompatibility, anti-inflammatory activity, antibiofilm efficacy, and antimicrobial potential, along with a sustained drug release profile. This formulation maintained favorable thermosensitive properties, suggesting its suitability for localized delivery applications. These findings indicate that incorporating curcumin into a chitosan-based hydrogel system may enhance its biological effectiveness and overcome some of the limitations associated with its conventional use. However, further *in vivo* and clinical investigations are required to confirm these observations and establish its potential role as an adjunct in periodontal therapy.

## Data Availability

The datasets generated and analyzed in this study are available from the corresponding author upon reasonable request.
